# Low-dose X-Ray induced genetic damage in human peripheral blood lymphocytes

**DOI:** 10.3389/fgene.2026.1768485

**Published:** 2026-02-25

**Authors:** Laura Camila Villalba-Rondón, Laura Vélez-Lemus, William Jaramillo-Garzón, Martín Pulido-Medellín, Nelson Rangel, Milena Rondón-Lagos

**Affiliations:** 1 Escuela de Ciencias Biológicas, Universidad Pedagógica y Tecnológica de Colombia, Tunja, Colombia; 2 Grupo de Física Médica, Universidad Nacional de Colombia, Bogotá, Colombia; 3 Grupo de Investigación en Medicina Veterinaria y Zootecnia (GIDIMEVETZ), Universidad Pedagógica y Tecnológica de Colombia, Tunja, Colombia; 4 Departamento de Nutrición y Bioquímica, Facultad de Ciencias, Pontificia Universidad Javeriana, Bogotá, Colombia

**Keywords:** chromosomal abnormalities, genotoxicity, low-dose radiation, micronuclei, x-rays

## Abstract

X-rays (XR) are electromagnetic waves capable of inducing significant biological effects in living organisms. Although widely used in medicine and industry, the impact of low-dose XR exposure on human health remains insufficiently characterized. XR can generate direct and indirect DNA damage such as single- and double-strand breaks, base modifications, and DNA–protein crosslinks, leading to chromosomal alterations that disrupt cellular homeostasis and may contribute to disease development. Although previous studies have reported general increases in cytogenetic damage at low exposures, they seldom provide detailed descriptions of which chromosomes are most affected, which structural or numerical alterations predominate, or how frequently each alteration occurs. This study aimed to characterize the type and frequency of chromosomal alterations and the spectrum of genetic damage, including both clonal and non-clonal alterations, in human lymphocytes exposed *in vitro* to a low X-ray dose (94.33 mGy), using non-exposed samples as controls. Peripheral blood was collected from 12 healthy donors, and genetic damage was assessed using GTG-banding cytogenetics and the cytokinesis-block micronucleus assay. Irradiated samples exhibited a significantly higher frequency of chromosomal alterations and fragile sites compared with their respective controls (p ≤ 0.0093). Among numerical alterations, monosomies were the most frequent, with chromosomes 8 and 21 being the most commonly affected, detected in 50% of irradiated samples. Structural chromosomal alterations predominantly involved chromosomes 11, 16, and 17, while recurrent deletions included del(15)(q22) and del(16)(q12). Among heterochromatic variants, chtb(9)(q12) was the most frequent, and fra(9)(q12) represented the most prevalent fragile site. MN frequency increased significantly after irradiation (p = 0.0214), and women exhibited higher MN frequencies than men regardless of treatment (p = 0.0224). Overall, these findings indicate that low-dose XR exposure is associated with detectable chromosomal damage and underscore the relevance of biosafety practices and cytogenetic monitoring approaches in contexts involving XR exposure, even at doses traditionally considered safe.

## 1 Introduction

XR are a form of ionizing radiation widely used in medical imaging, industrial testing, and research applications. During diagnostic procedures, both patients and healthcare personnel may be exposed to low doses of XR (<100 mGy). The ionization produced by this type of radiation can induce physical, chemical, and biological damage in living organisms. Historically, nuclear events such as those in Hiroshima, Nagasaki, and Chernobyl have revealed the devastating effects of ionizing radiation on human health, including skin burns, ocular lens damage, thyroid dysfunction, and an increased risk of cancer, particularly leukemia (UNSCEAR, 2020). The biological effects of ionizing radiation have been extensively studied for decades, with most research focused on high-dose exposures. Although the detrimental consequences of high-dose radiation are well established, the biological impact of low-dose XR exposure on chromosomal integrity remains less clear and continues to be a matter of debate, as contradictory findings persist (BEIR VII, 2006; WHO, 2023). Several studies have shown that low-dose XR can induce DNA double-strand breaks, MN formation, sister chromatid exchanges, and other chromosomal abnormalities, suggesting that even minimal exposure may compromise genome stability (Huang et al., 2003; Rothkamm and Löbrich, 2003). However, other investigations report negligible or transient effects, proposing the existence of efficient DNA repair mechanisms or adaptive responses that mitigate chromosomal damage at low doses (Tubiana et al., 2006). These inconsistencies highlight the need for more comprehensive and standardized studies to clarify the true chromosomal consequences of low-dose XR exposure. A deeper understanding of these subtle yet potentially cumulative effects is essential for accurately assessing long-term health risks and refining radioprotection strategies aimed at safeguarding both, patients and occupationally exposed individuals (INSST, 2025).

Several studies have documented the harmful health effects of XR exposure, leading to the establishment of annual dose limits for individuals with occupational exposure, such as nuclear power plant workers (ICRP, 2007). Notably, epidemiological evidence indicates that children exposed to multiple computed tomography scans show an increased risk of developing leukemia and brain tumors (Henry and Arcangeli, 2021).

Despite these findings, a substantial knowledge gap persists regarding the biological consequences of low-dose XR exposure. In particular, there is limited consensus on the types of chromosomal alterations most reliably induced at low doses (e.g., losses, gains, dicentrics, translocations, deletions, MN, or locus-specific breaks) and on their frequency, which appears highly variable across studies (Rothkamm and Löbrich, 2003; Smith et al., 2003). Inconsistencies arise from differences in detection methods, dose-rate conditions and cell types examined, making it difficult to determine whether low-dose XR induce chromosomal alterations detectable through sensitive cytogenetic assays (Bryant et al., 2010). This gap is particularly relevant given that the general population is continuously exposed to ionizing radiation, both, from natural sources, such as cosmic rays, terrestrial radiation, and radionuclides contained in minerals, and from artificial sources, including XR-emitting equipment used in diagnostic and therapeutic procedures (NCRP, 2009).

The limited understanding of the genetic and chromosomal risks associated with low-intensity exposures hinders accurate risk assessment and the development of effective radioprotection strategies, as most low-dose studies report general increases in cytogenetic damage without detailing the affected chromosomes, the predominant types of alterations, their clonal or non-clonal nature, or their frequency. This is particularly relevant because even low-dose exposures may generate cumulative genetic damage and increase the risk of chronic diseases, including cancer. Therefore, a detailed characterization of the type, frequency, and spectrum of chromosomal alterations induced by low-dose XR exposure is essential to better elucidate its biological effects and to support public health protection strategies.

In this context, the present study aimed to characterize the cytogenetic effects of low-dose XR exposure in human peripheral blood lymphocytes *in vitro*, focusing on the type and frequency of numerical and structural chromosomal alterations, including both clonal and non-clonal events, and the overall spectrum of genetic damage, using non-exposed samples as controls. Our findings demonstrate that even low-dose XR exposure can induce numerical and structural chromosomal alterations, increase MN frequency, and enhance chromosomal fragility. These results provide important insights into the biological consequences of low-dose radiation and underscore the need to strengthen biosafety measures and radioprotection strategies for individuals exposed to XR, even at doses traditionally considered safe.

## 2 Materials and methods

### 2.1 Study population

The study included twelve (12) healthy volunteers over 18 years of age, divided into two age groups. Group 1 comprised three (3) men and three (3) women aged 20–25 years, while Group 2 comprised three (3) men and three (3) women aged 26–30 years (Table 1). Exclusion criteria included a history of occupational or recent medical exposure to XR, as well as the presence of medical conditions such as liver disease, diabetes mellitus, anemia, thyroid disorders, cancer, or epilepsy.

**TABLE 1:** Distribution of participants by age group.

Demographic characteristics	Age group 1 (20–25 years)	Age group 2 (26–30 years)
Number	*6*	6
Age (mean ± SD)	22.5 +/- 1.6	27.2 +/- 1.6
Male (n)	3	3
Female (n)	3	3

SD, standard deviation.

The study was conducted in accordance with the Declaration of Helsinki and was approved by the Ethics Committee of the Universidad Pedagógica y Tecnológica de Colombia (approval date: 12 September 2024). Written informed consent was obtained from all participants.

### 2.2 Sample collection

Peripheral blood samples were collected from the twelve (12) participants by venipuncture into three (3) 5-mL Vacutainer® tubes containing sodium heparin (BD Ref. 367874). Standardized harvest protocols were applied.

### 2.3 Determination of the X-rays dose for *in vitro* blood sample irradiation

To determine the minimum dose at which cytogenetic alterations in blood cells become detectable, eight peripheral blood samples from the same donor were irradiated *in vitro* with XR, each receiving a distinct dose: 2.8 mGy, 7.8 mGy, 9.9 mGy, 15.1 mGy, 22.6 mGy, 47.8 mGy, 72.5 mGy, or 94.3 mGy. These doses correspond to typical exposure levels encountered by patients and healthcare workers during medical diagnostic imaging procedures (Katsura et al., 2016). During irradiation, the blood samples were positioned at the center of the XR beam generated by an industrial XR unit (ICM SITEX) operated at 170 kV for 2.1 min. To replicate the irradiation conditions of a standard adult in an anteroposterior (AP) projection, a polymethyl methacrylate (PMMA) acrylic plate (30 × 30 cm, 10 mm thick) was used to simulate soft tissue, approximating the depth of the first major blood vessel (the internal thoracic artery) in the AP projection. The entrance dose for each sample was measured using a PTW-DIADOS E dosimetric system.

Based on this standardization process, a dose of 94.3 mGy was selected for subsequent sample irradiation. This dose falls within the low-dose XR range, yet is sufficiently high to induce early cytogenetic responses, as doses in the range of ∼50–100 mGy have been consistently shown to reliably generate detectable DNA double-strand breaks in human cells (Jain et al., 2023; Rothkamm and Löbrich, 2003; Yamashita and Suda, 2021), as well as low-frequency of chromosomal alterations and MN measurable by standard cytogenetic assays (Mitchell and Norman, 1987; Shimura and Kojima, 2018; Yamashita and Suda, 2021). At this level, radiation exposure remains radiologically safe while providing a biologically informative window in which subtle radiation-induced chromosomal alterations can be detected and characterized. Therefore, 94.3 mGy was selected as an appropriate reference dose for evaluating the impact of low-dose XR on chromosomal integrity.

### 2.4 Cytogenetic analysis (GTG-banding)

GTG-banding chromosome analysis was applied to evaluate numerical and structural chromosomal alterations induced by low-dose XR exposure in human lymphocytes. This technique enables the identification of all types of numerical and structural alterations, including deletions, translocations, inversions, dicentric and ring chromosomes. The use of GTG-banding provides a comprehensive visualization of radiation-induced damage across the entire karyotype, allowing precise classification of both, classical radiation markers (dicentric and ring chromosomes) and additional structural rearrangements within a single analytical framework.

Metaphases were obtained using standard harvesting protocols for GTG banding. Briefly, both irradiated and non-irradiated peripheral blood samples, were cultured in duplicate using RPMI 1640 medium (Sigma, St. Louis, MO, United States), supplemented with 10% fetal bovine serum (Sigma, St. Louis, MO, United States), and 150 µL of phytohemagglutinin M (Gibco, Life Technologies, Waltham, MA, United States). Cultures were incubated at 37 °C in a humidified atmosphere containing 5% CO_2_ for 72 h. Bromodeoxyuridine (BrdU) was not added to the culture medium. After incubation, colchicine (0.03 μg/mL; Sigma) was added for 30 min to arrest cells in metaphase. The samples were then subjected to hypotonic treatment with 0.075 M KCl and fixed three times in freshly prepared Carnoy’s fixative (methanol:acetic acid, 3:1).

Chromosomal spreads were prepared and GTG-banded using 0.25% trypsin (Gibco, Life Technologies, Waltham, MA, United States), followed by Giemsa staining (Sigma, St. Louis, MO, United States). Metaphase analysis, image capture, and karyotype assembly were performed using an Olympus microscope equipped with Cytovision System 7.4 software (Leica Biosystems, Richmond, IL, United States). For each sample (irradiated and non-irradiated), between 20 and 25 well-spread metaphases with optimal morphology were analyzed, selected from at least two different slides.

Chromosomal alterations (CAs), including both numerical and structural aberrations, as well as chromosomal variants (CVs) such as chromatid breaks (chtb) and chromosomal breaks (chrb), fragilities (fra) and heterochromatin increased on the long arm of chromosomes 9 (9qh+) and 16 (16qh+), were evaluated in a total of 586 metaphases (295 from irradiated and 291 from non-irradiated samples). All CAs and CVs were classified according to the International System for Human Cytogenomic Nomenclature 2024 (ISCN) (Hastings et al., 2024). Fragile sites were identified in GTG-banded metaphases as recurrent chromosomal gaps, constrictions, or non-staining regions affecting one or both chromatids without complete chromosomal breakage. Fragilities were reported following the ISCN 2024 (Hastings et al., 2024).

### 2.5 Cytokinesis-block micronucleus (CBMN) assay

The CBMN assay was performed according to the standardized protocol described by Fenech (2007). Briefly, peripheral blood samples from each participant, both irradiated and non-irradiated, were cultured in duplicate in 5 mL of RPMI 1640 medium (Sigma), supplemented with 10% fetal bovine serum (Sigma) and 150 µL of phytohemagglutinin M (Gibco). The cultures were incubated at 37 °C in a humidified atmosphere containing 5% CO_2_ for 44 h. Subsequently, cytochalasin B (Sigma) was added at a final concentration of 5 μg/mL, and incubation continued until a total culture time of 72 h was reached.

After incubation, the cells were subjected to hypotonic treatment with 0.075 M KCl for 8 min, followed by fixation in Carnoy’s solution (methanol:acetic acid, 3:1). Fixed cell suspensions were spread onto clean glass slides and stained with 5% (v/v) Giemsa for 12 min.

MN, nucleoplasmic bridges (NPB), and nuclear buds (NBUD) were identified in 1,000 binucleated cells per sample (irradiated and non-irradiated). Image acquisition and scoring were performed using an Olympus microscope equipped with Cytovision System 7.4 software (Leica Biosystems, Richmond, IL, United States). The identification of MN, NPB, and NBUD was based on the morphological criteria established by Fenech (2000).

### 2.6 Statistical analysis

All cytogenetic parameters were analyzed to compare irradiated and non-irradiated samples. GTG-banding data were evaluated using Fisher’s exact test, Student’s t-test, and the unpaired Mann–Whitney–Wilcoxon test to account for parametric and non-parametric distributions. Data normality and homogeneity of variances were assessed with the Shapiro–Wilk and Bartlett tests, respectively. The effects of irradiation status (irradiated vs. non-irradiated), sex (male vs. female), and their interaction on the frequencies of MN, NPB, and NBUD were assessed using a two-way ordinary ANOVA, with assumptions of normality and variance homogeneity verified prior to analysis. Paired *t*-tests were applied to determine whether differences between irradiated and non-irradiated samples were statistically significant. All analyses were performed using GraphPad Prism seven and RStudio (version 4.0.2). Data are presented as mean ± standard error of the mean (SEM), and statistical significance was defined at *p* ≤ 0.05, with additional thresholds of *p* ≤ 0.01 and *p* ≤ 0.001 where appropriate.

## 3 Results

### 3.1 Exposure to low doses of XR induces numerical and structural chromosomal alterations

Cytogenetic analysis using GTG-banding was performed on both irradiated and non-irradiated samples, revealing a consistent diploid modal number (2n) in all cases. According to international recommendations for constitutional cytogenetic studies (CCMG-CCGM National Office, 2021; Ozkan and Lacerda, 2023), the analysis of 10–20 metaphases is generally considered sufficient to obtain reliable cytogenetic information. In the present study, the evaluation of 25 metaphases per individual was selected to allow a descriptive characterization of numerical and structural chromosomal alterations, including both clonal and non-clonal events, without any biodosimetric purpose. This approach is consistent with experimental cytogenetic studies assessing radiation-induced chromosomal aberrations rather than dose estimation (Kote-Jarai et al., 2006).

In total, 586 metaphases were examined (295 from irradiated and 291 from non-irradiated samples), all of which exhibited adequate morphology and high-quality chromosomal spreading. The small difference in the number of analyzed metaphases between groups is attributable to inter-individual variation in the mitotic index among the samples included in the study.

**FIGURE 1:**
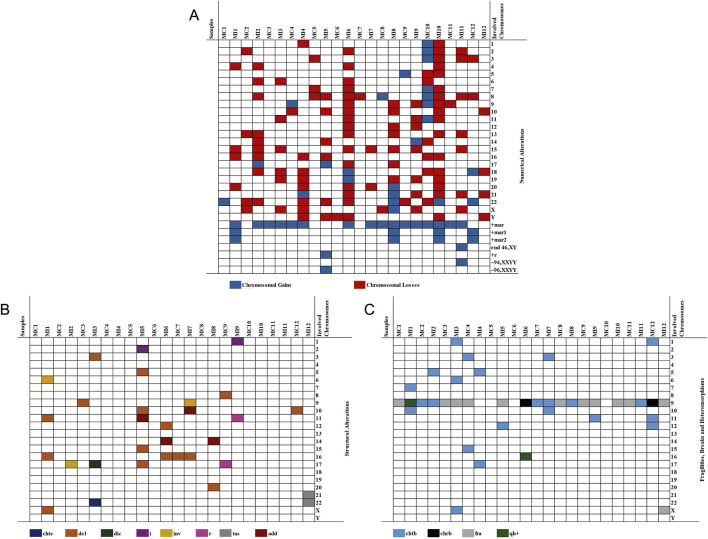
Numerical and structural chromosomal alterations and heteromorphisms in irradiated (MI) and corresponding non-irradiated control samples (MC). **(A)** Numerical chromosomal alterations. **(B)** Structural chromosomal alterations. **(C)** Chromosomal heteromorphisms. Each column represents an individual sample (irradiated or control), and each row corresponds to a chromosome exhibiting an alteration or heteromorphism. The type of alteration is indicated by the color code shown at the bottom of the figure. end, endoreduplication; chte, chromatid exchange; del, deletion; dic, dicentric chromosome; i, isochromosome; inv, inversion; r, ring chromosome; tas, telomeric associations; add, additional material of unknown origin; chtb, chromatid breaks; chrb, chromosomal breaks; fra, fragile sites; qh+, increased heterochromatin in the long arm.

A significantly higher frequency of chromosomal alterations (CA) and fragile sites (fra) was observed in irradiated samples (269 alterations) compared to their respective non-irradiated controls (174 alterations) (p ≤ 0.0093; unpaired Mann–Whitney–Wilcoxon test) (Table 2; Figure 1).

**TABLE 2:** Frequencies and percentages of chromosomal alterations (CAs) and chromosomal variants (CVs) identified in irradiated (MI) and non-irradiated (MC) lymphocyte samples.

CAs and CVs	Number of alterations		
​	MI n (%)	MC n (%)	p
NCAs	190 (64.4)	44 (15.12)	0.0001**
Monosomies	138 (46.78)	20 (6.87)	0.0001**
Trisomies	49 (16.61)	24 (8.24)	0.0025*
Polyploidies	3 (1.01)	0 (0)	0.2488
SCAs	28 (9.5)	5 (1.71)	0.0001**
chtb/chrb	18 (6.1)	11 (3.78)	0.2531
fra	3 (1.01)	0 (0)	0.2488
fra(9)(q12)	28 (9.5)	14 (4.81)	0.0364*
Heteromorphisms	2 (0.68)	0 (0)	0.4992
Total alterations	269	74	0.0001**
Mean	33.63	9.25	​
SD	45.2	9.51	​

MI, irradiated samples; MC, non-irradiated control samples; CAs, chromosomal alterations; CVs, chromosomal variants; NCAs, numerical chromosomal alterations; SCAs, structural chromosomal alterations; chtb, chromatid break; chrb, chromosomal break; fra, fragilities; fra(9)(q12), fragility in the long arm of chromosome 9, region 1, band 2; SD, standard deviation. *Statistically significant differences compared with non-irradiated control samples at p ≤ 0.05. **Statistically significant differences compared with non-irradiated control samples at p ≤ 0.01 (Fisher’s exact test).

In the irradiated samples, 295 metaphases were analyzed, identifying 190 numerical chromosomal alterations (NCAs) and 28 structural chromosomal alterations (SCAs), present in all evaluated cases (100%). Additionally, 18 chromatid or chromosomal breaks (chtb/chrb) were detected in 92% of the samples, along with two chromosomal heteromorphisms (9qh+ and 16qh+) and 31 fragile sites (fra) in 83.3% of the samples. Among the NCAs, monosomies were the most frequent (46.78%), followed by trisomies (16.61%) and polyploidies (1.01%). Chromosomes 8 and 21 were the most frequently affected by monosomies (8.7%), detected in 50% of irradiated samples. In contrast, most trisomies corresponded to marker chromosomes (63.5%), observed in 92% of cases (Figure 1).

A total of 28 SCAs were identified in all irradiated samples (100%) (Table 2). The most frequent SCAs were deletions (50%), followed by additional material of unknown origin (add, 14.28%), inversions (inv, 10.71%), isochromosomes (i, 7.14%), and ring chromosomes (r, 7.14%). Less common alterations included dicentric chromosomes (dic), chromatid exchanges (chte), and telomeric associations (tas), each with a frequency of 3.57%. Chromosomes 11, 16, and 17 were the most frequently involved in SCAs. Among deletions, del(15)(q22) and del(16)(q22) were observed in two irradiated samples (16.66%). Chromatid and chromosomal breaks (chtb/chrb) were observed in all irradiated samples, with chtb(9)(q12) being the most frequent (16.66%), present in three samples (25%).

Analysis of chromosomal fragility revealed a higher frequency of chromosomal fragilities in irradiated samples compared with non-irradiated controls. Fragilities were identified as recurrent gaps or constrictions observed in GTG-banded metaphases and were classified according to ISCN nomenclature (ISCN 2024). Among the detected fragile sites, fra(9)(q12) was the most prevalent, observed in 83.3% of the irradiated samples (90.32% of total identified fra). Other fragile regions were detected sporadically and at markedly lower frequencies.

A total of 291 metaphases were analyzed in the non-irradiated control samples. 44 numerical NCAs were identified in all samples (100%), whereas 5 SCAs were detected in 33.3% of the samples (Table 2). Additionally, 11 chtb/chrb were observed in 33.3% of the samples, while 14 fra(9)(q12) were detected in 66.6% of the samples. Among the NCAs, trisomies were the most frequent (8.24%), followed by monosomies (6.87%). Chromosome X was the most commonly affected by monosomies, detected in 16.6% of the control samples. Most trisomies corresponded to marker chromosomes (33.3%), present in 58.3% of the samples.

Regarding SCAs, a total of 5 alterations were identified, of which 80% were deletions, present in 25% of the control samples. Additionally, one ring chromosome was detected. The deletions involved chromosomes 8, 9, 10, 16, and 17. chtb/chrb were observed in 33.3% of the samples, with chtb(9)(q12) being the most frequent (27.27%), detected in three samples (16.6%). Concerning chromosomal fragilities, the only one identified was fra(9)(q12), observed in 66.6% of control samples, with an overall frequency of 4.81%.

Comparison of chromosomal variants (CVs), NCAs, and SCAs between irradiated samples and their paired non-irradiated controls revealed statistically significant differences in 83.33% of the cases (p ≤ 0.02; Fisher’s exact test) (Table 3).

**TABLE 3:** Comparative frequencies of chromosomal alterations (CAs) and variants (CVs) in paired irradiated (MI) and non-irradiated control (MC) lymphocyte samples.

No	MI						No	MC						p
	NCA	SCA	fra	chrb/chtb	CV	Total		NCA	SCA	fra	chrb/chtb	CV	Total	
1	10	4	4	2	1	21	1	1	0	1	0	0	2	0.0001**
2	13	1	1	2	0	17	2	4	0	0	1	0	5	0.0150**
3	12	3	1	4	0	20	3	1	1	1	0	0	3	0.0004**
4	17	0	0	2	0	19	4	3	0	2	2	0	7	0.0256*
5	15	7	3	1	0	26	5	1	0	0	0	0	1	0.0001**
6	34	3	2	1	1	41	6	1	0	0	0	0	1	0.0001**
7	3	4	6	3	0	16	7	1	1	2	1	0	5	0.0241*
8	18	2	1	1	0	22	8	4	0	0	0	0	4	0.0004**
9	11	2	3	1	0	17	9	4	2	2	0	0	8	0.1004
10	38	0	1	0	0	39	10	17	0	0	0	0	17	0.0029**
11	13	0	7	1	0	21	11	2	0	1	0	0	3	0.0002**
12	6	1	3	0	0	10	12	6	1	4	7	0	18	0.1245
TOTAL	89	27	32	18	2	269	TOTAL	45	5	13	11	0	74	​

MI, irradiated samples; MC, non-irradiated control samples; NCAs, numerical chromosomal alterations; SCAs, structural chromosomal alterations; fra, fragilities; chtb, chromatid break; chrb, chromosomal break; CVs, chromosomal variants. Statistically significant differences based on the total number of chromosomal alterations (CAs) and variants (CVs) per sample compared with non-irradiated controls at p*≤0.05; **at p ≤ 0.01 (Fisher’s exact test). The total number of metaphases analysed in the irradiated samples was 295, while in the non-irradiated control samples it was 291.

### 3.2 Exposure to low doses of XR induces DNA damage

**FIGURE 2:**
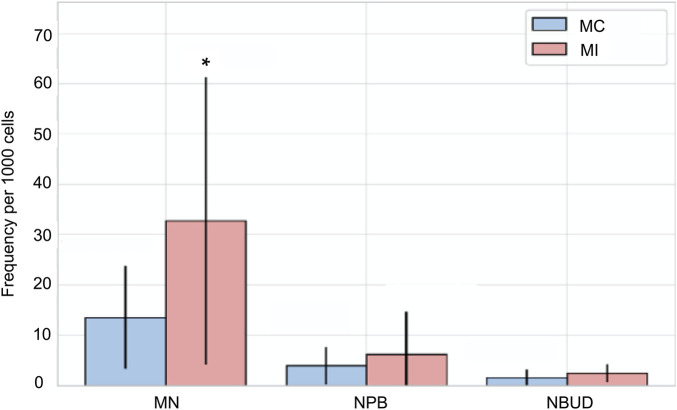
Frequency of micronuclei (MN), nucleoplasmic bridges (NPB), and nuclear buds (NBUD) in irradiated samples (MI) and their corresponding non-irradiated controls (MC). Error bars represent the standard deviation of counts obtained from 1,000 binucleated cells per sample. *Statistically significant differences compared with control samples (p ≤ 0.05; unpaired Mann–Whitney–Wilcoxon test).

**FIGURE 3:**
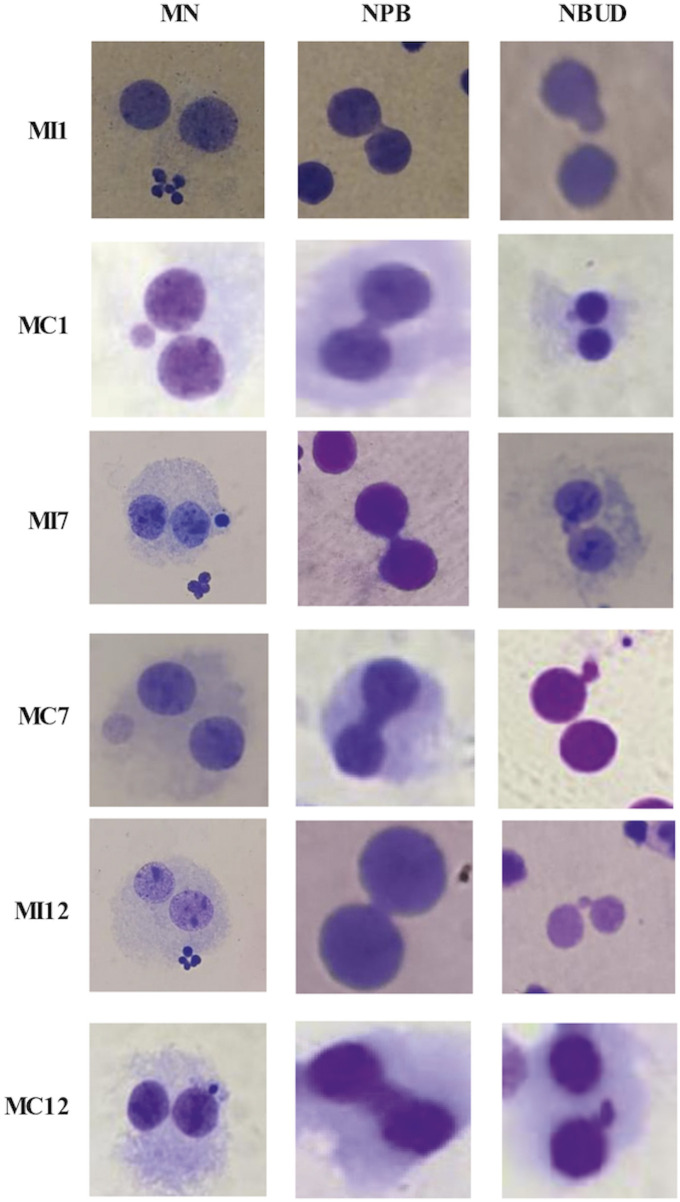
Representative images of micronuclei (MN), nucleoplasmic bridges (NPB), and nuclear buds (NBUD) in irradiated samples (MI) and their corresponding non-irradiated control samples (MC). Abbreviations: MI1, irradiated sample 1; MC1, non-irradiated control sample 1; MI7, irradiated sample 7; MC7, non-irradiated control sample 7; MI12, irradiated sample 12; MC12, non-irradiated control sample 12.

To assess the frequency of MN, nucleoplasmic bridges (NPB), and nuclear buds (NBUD), 1,000 binucleated cells were analyzed per sample in both, irradiated and corresponding non-irradiated controls, totaling 24,000 cells examined. Individual MN frequencies for irradiated samples and corresponding non-irradiated controls, are presented for each donor, revealing consistent interindividual variability (Table 4). MN frequencies were higher in irradiated samples than in non-irradiated controls in 11 out of 12 donors. Overall, irradiated samples exhibited a statistically significant increase in MN frequency compared with their respective non-irradiated controls (32.8 ± 28.6 vs. 13.5 ± 10.2; p ≤ 0.05; unpaired Mann–Whitney–Wilcoxon test) (Figures 2, 3).

**TABLE 4:** Individual micronuclei (MN) frequencies in irradiated samples (MI) and their corresponding non-irradiated controls (MC).

No	MI (n)	MC (n)
1	87	11
2	96	44
3	24	12
4	13	10
5	25	14
6	10	5
7	37	10
8	19	13
9	26	13
10	15	3
11	12	14
12	29	13
TOTAL	393	162

Individual MN frequencies for irradiated samples and corresponding non-irradiated controls, are presented for each donor, revealing consistent interindividual variability. MN, frequencies were higher in irradiated samples than in non-irradiated controls in 11 out of 12 donors.

In contrast, no statistically significant differences were observed in the frequencies of NPB or NBUD between the two groups. NPB frequencies were 6.2 ± 8.5 in the irradiated samples and 3.9 ± 3.8 in the non-irradiated controls, while NBUD frequencies were 2.4 ± 1.8 in the irradiated samples and 1.6 ± 1.5 in the non-irradiated controls (Figures 2, 3).

### 3.3 Sex as determinant of genetic damage: comparison between irradiated and non-irradiated samples

To assess whether irradiation status and sex, influenced the frequencies of MN, NPB and NBUD, a two-way ordinary ANOVA was performed. The analysis revealed no significant interaction between irradiation status and sex (*p* = 0.1432), indicating that the effect of irradiation on these cytogenetic endpoints was independent of sex.

However, a significant main effect of irradiation status was observed (*p* = 0.0214), with irradiated individuals exhibiting higher MN frequencies compared with non-irradiated controls. A significant main effect of sex was also detected (*p* = 0.0224), with women showing higher MN frequencies than men in both irradiated and non-irradiated samples. Consistent trends were observed for NPB and NBUD, although these effects did not reach statistical significance (data not shown).

**FIGURE 4:**
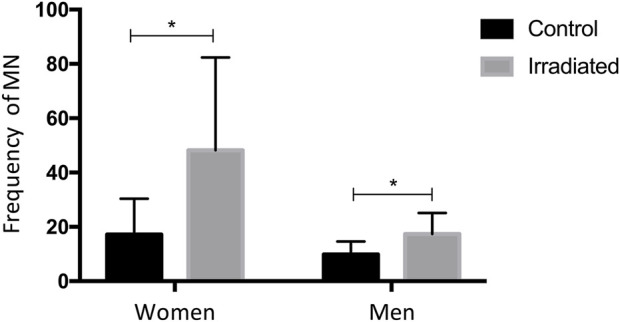
Micronuclei (MN) frequencies in non-irradiated vs. irradiated samples by sex. MN counts were significantly higher in irradiated samples compared with their corresponding controls. Women exhibited higher MN frequencies than men in both, control and irradiated samples. Data are expressed as mean ± standard deviation. *Statistically significant differences compared with control samples (*p* ≤ 0.05, two-way ANOVA and paired *t*-test).

Overall, these findings indicate that irradiation increases MN frequency and that sex-related differences exist in cytogenetic responses, with women displaying higher MN frequencies than men regardless of irradiation status This pattern suggests that women may be more susceptible to genomic instability, both at baseline and following radiation exposure (Figure 4).

## 4 Discussion

Ionizing radiation is an unavoidable component of the human environment, arising from both natural and anthropogenic sources, with medical X-rays representing the primary source of artificial exposure for the general population (Karmaker et al., 2021; Jackson and Bartek, 2009; UNSCEAR, 2020). The widespread and increasing use of diagnostic and interventional procedures has made medical XR the dominant man-made contributor to annual effective dose in many countries (Betlazar et al., 2016; ICRP, 2007). Although typically delivered at low doses, XR exposure can induce subtle yet persistent molecular and cellular effects, particularly in radiosensitive systems such as the hematopoietic compartment, leading to genome instability, impaired DNA repair, and oxidative and inflammatory responses (Cherednichenko et al., 2024). Despite their extensive clinical application, the subclinical and long-term genotoxic effects of low-dose XR exposure remain insufficiently characterized, underscoring the need for early detection of genetic alterations to refine radiobiological risk models and strengthen radioprotection strategies (Gnanasekaran, 2021).

In this context, a substantial knowledge gap persists regarding the type and frequency of chromosomal alterations induced by low-dose XR. Although previous studies have shown general increases in cytogenetic damage at low exposures, they rarely provide detailed descriptions of which chromosomes are most affected, which structural or numerical alterations predominate, or how frequently each alteration occurs. To address this limitation, our study employed GTG-banding to perform an *in vitro* cytogenetic assessment focused on the characterization of radiation-induced chromosomal damage, enabling the visualization of both clonal and non-clonal chromosomal abnormalities and providing precise information on their frequency.

Our results demonstrate that low-dose XR exposure induces measurable genetic and chromosomal damage in human peripheral blood lymphocytes *in vitro*, as reflected by increased MN formation and higher frequencies of structural and numerical chromosomal alterations associated with chromosomal instability. These findings are consistent with radiation-induced disruptions of DNA integrity and chromosomal segregation, indicative of persistent genotoxic stress. Similar cytogenetic effects have been reported in occupationally exposed populations, such as interventional radiologists chronically exposed to higher cumulative doses of ionizing radiation (Kochanova et al., 2023). Although differences in exposure patterns preclude direct dose–response comparisons, our data show that a substantially lower acute dose (94.3 mGy) is sufficient to induce significant chromosomal alterations and fragile sites compared with non-irradiated controls, reinforcing the capacity of ionizing radiation to induce cytogenetic damage.

Importantly, our results suggest that measurable chromosomal instability can occur at doses well below those reported in occupational settings, underscoring the sensitivity of cytogenetic endpoints for detecting radiation-induced genetic damage and supporting their utility in biomonitoring and radiobiological risk assessment.

In this study, monosomies were the most frequent NCAs detected after low-dose XR exposure, likely reflecting radiation-induced disruption of mitotic spindle function and cell cycle control, processes sensitive to oxidative stress (Bakhoum et al., 2015). Such alterations can lead to chromosome missegregation and persistent chromosomal instability across cell generations (Little, 2000). Chromosomes 8 and 21 were most frequently affected, appearing in 50% of irradiated samples, which suggests intrinsic chromosomal vulnerability (Bonassi et al., 2006; Mitelman et al., 2007). These findings are consistent with radiation-induced genomic instability, characterized by the persistence and accumulation of chromosomal alterations over time (Bonassi et al., 2006).

A markedly higher number of SCAs was detected in irradiated samples (28) compared with non-irradiated controls (5), underscoring the high radiosensitivity of human lymphocytes even at low XR doses. The predominant SCAs including: deletions, inversions, isochromosomes, and ring chromosomes, are consistent with radiation-induced DNA double-strand breaks (DSBs), one of the most deleterious consequences of radiation-induced damage. SCAs mainly involved chromosomes 11, 16, and 17, which contain regions enriched in genes related to cell cycle regulation and DNA damage response, potentially increasing their susceptibility to misrepair following irradiation (Goodarzi and Jeggo, 2013; Obe et al., 2002). Recurrent deletions: del(15)(q22) and del(16)(q22) further support the presence of chromosomal regions prone to radiation-induced breakage, possibly influenced by chromatin organization and replication timing (Wang et al., 2020).

In addition, the high frequency of the heterochromatic variant chtb(9)(q12) underscores the sensitivity of constitutive heterochromatin to radiation-induced damage. Pericentromeric regions such as 9q12, enriched in repetitive DNA, are particularly prone to fragility under replication stress and impaired DNA repair, a vulnerability that may be exacerbated by ionizing radiation (Brown and Freudenreich, 2021). These findings support the notion that radiation-induced chromosomal damage is shaped by chromosome-specific features and chromatin architecture rather than being randomly distributed across the genome (Obe et al., 2002; Bickmore and van Steensel, 2013).

Moreover, low-dose XR exposure induced a marked rise in chromosomal fragilities in peripheral blood lymphocytes, which are recognized as sensitive biomarkers of early radiation-induced DNA damage (Amundson et al., 2001). Notably, fra(9)(q12), a rare heritable fragile site associated with specific DNA sequence features, was the most prevalent site, detected in 83.3% of irradiated samples, highlighting its particular sensitivity to low-dose XR (Feng and Chakraborty, 2017). Unlike common fragile sites, which are broadly present and primarily induced by replication stress, fra(9)(q12) reflects site-specific genomic vulnerability that may be exacerbated by ionizing radiation. These findings are consistent with evidence that low-dose radiation can produce subtle yet cumulative genotoxic effects with potential long-term carcinogenic implications (Mothersill and Seymour, 2014).

The markedly lower frequency of chromosomal fragilities, NCAs and SCAs in non-irradiated samples, suggest that XR exposure acts as a direct inducer of chromosomal damage under the experimental conditions evaluated. This evidence underscores the biological relevance of even minimal radiation exposure, as repeated low-dose events in clinical or occupational contexts, may exert cumulative genotoxic effects with potential long-term health implications.

Dicentric chromosomes, although highly specific biomarkers of ionizing radiation–induced damage, were not detected in this study. This is consistent with their intrinsic instability and strong negative selection during mitosis, which limits their persistence at metaphase, particularly at low radiation doses where their expected frequency approaches background levels (∼1/1,000 cells) (Wilkins et al., 2008; Lee Y. et al., 2019). Reliable dicentric detection typically requires first-division metaphases obtained from 48-h cultures with cell-cycle discrimination, conditions not applied here, as lymphocytes were cultured for 72 h without BrdU to enable the characterization of both stable and unstable chromosomal alterations (Lee Y. et al., 2019). Under these conditions, dicentrics are likely eliminated across divisions or repaired through pathways such as non-homologous end joining or homologous recombination (Murmann-Konda et al., 2021). Accordingly, their absence aligns with both the low-dose exposure and the experimental design. To address this limitation, the MN assay was included as a complementary endpoint, allowing detection of unstable chromosomal damage that may not persist to metaphase.

Consistent with the chromosomal alterations observed in irradiated samples, MN frequency was significantly increased compared with non-irradiated controls, underscoring the sensitivity of the MN assay for detecting radiation-induced genomic damage at low doses. This finding confirms that ionizing radiation induces measurable genetic damage, in line with previous evidence showing that radiation promotes DNA strand breaks, acentric fragment formation, and subsequent MN generation during mitosis (Durante and Formenti, 2018). As such, MN formation represents a robust indicator of radiation-induced genomic instability and impaired DNA repair (Fenech, 2000; Holland et al., 2008; Wilhelm et al., 2020).

Additionally, MN frequency was significantly influenced by sex, with women exhibiting higher levels than men regardless of irradiation status. This difference may reflect hormonal influences, particularly estrogen-mediated effects on redox balance and genomic stability, as well as sex-related differences in oxidative damage repair capacity (Schmitz-Feuerhake et al., 2016; Topic et al., 2016; Narendran et al., 2019). The sex-specific increase in MN frequency after irradiation was not accompanied by a parallel rise in metaphase-detected chromosomal alterations, highlighting the distinct sensitivities of these cytogenetic endpoints. While metaphase analysis identifies alterations that persist through cell-cycle progression, the MN assay captures both chromosome breakage and whole-chromosome loss occurring during previous mitotic events, thereby reflecting earlier or transient genomic instability (Fenech, 2007; Norppa and Falck, 2003). Conversely, more stringent cell-cycle checkpoint control and more efficient elimination of heavily damaged cells in female lymphocytes may limit the progression of cells harboring complex aberrations to mitosis, resulting in lower detectable chromosomal alteration frequencies despite elevated MN levels (Rall-Scharpf et al., 2021; Broestl and Rubin, 2021). Collectively, these findings highlight the complementary nature of MN and chromosomal alteration assays and underscore the importance of considering sex as a key biological variable in radiobiological research and radiation risk assessment (Ji et al., 2019; ICRP, 2017).

Cytogenetic analyses, particularly the MN assay, offer a sensitive and reliable approach for detecting early radiation-induced genetic alterations and serve as biomarkers of both, exposure and effect (Fenech, 1993). These tools are especially valuable for occupational and environmental monitoring, including healthcare workers, chronically exposed professionals, patients undergoing radiotherapy, and populations living near radiation sources (Gnanasekaran, 2021; IAEA, 2014; Monteiro Gil et al., 2020). Early detection of cytogenetic damage supports preventive interventions, improves disease risk estimation, and informs evidence-based public health strategies.

Although the adverse health effects of high-dose radiation are well established and underpin current ICRP-based protection frameworks (Stewart et al., 2012), the biological consequences of chronic low-dose occupational exposure remain insufficiently characterized, particularly using genetic damage biomarkers (Mbutu-Austin et al., 2025). In this context, our findings demonstrate that low-dose XR exposure induces detectable chromosomal alterations, fragile sites, and MN formation in human lymphocytes, reinforcing the sensitivity of cytogenetic endpoints for monitoring radiation-induced effects.

Collectively, these results support the integration of cytogenetic biomarkers into radiation protection and biomonitoring strategies, especially for chronically or occupationally exposed individuals. Chromosomal alterations and MN formation are established indicators of genomic instability and have been associated with increased disease risk, including cancer, as supported by epidemiological evidence from radiation workers and atomic bomb survivors (Piotrowski et al., 2017). The detection of chromosomal damage at low XR doses further indicates that genome integrity may be compromised even at exposure levels traditionally considered low. Although adaptive responses such as inducible DNA repair have been described in lymphocytes (Kelsey et al., 1991), these mechanisms do not preclude initial damage induction, highlighting the complexity of low-dose radiation responses and the need to better understand how damage, repair capacity, and individual susceptibility interact to inform radioprotection frameworks.

## 5 Conclusion

Low-dose XR exposure was found to induce chromosomal alterations and increase MN formation in human lymphocytes, confirming the high sensitivity of these cells to ionizing radiation. Importantly, the detailed characterization of the type and frequency of chromosomal alterations, encompassing both clonal and non-clonal events, provides refined evidence of how low-dose exposure compromises genome integrity. By delineating specific cytogenetic signatures associated with low-dose XR exposure scenarios, this work fills a critical gap in understanding the biological impact of such exposures and contributes essential data for strengthening radiobiological risk assessments and radioprotection frameworks. Furthermore, sex-related differences in MN frequency indicate that intrinsic biological factors may modulate susceptibility to radiation-induced genetic damage, underscoring the complexity of cellular responses to genotoxic stress. Overall, the findings highlight the value of cytogenetic assays as sensitive tools for biomonitoring radiation effects and support their potential to inform risk assessment, guide preventive strategies, and contribute to public health efforts aimed at minimizing long-term radiation-related health risks.

## Data Availability

The original contributions presented in the study are included in the article/supplementary material, further inquiries can be directed to the corresponding authors.

## References

[B1] Amundson S. A. Bittner M. Meltzer P. Trent J. Fornance A. J. Jr. (2001). Biological indicators for the identification of ionizing radiation exposure in humans. Expert Rev. Mol. Diagnostics 1, 211–219. 10.1586/14737159.1.2.211 11901816

[B2] Bakhoum S. F. Kabeche L. Wood M. D. Laucius C. D. Qu D. Laughney A. M. (2015). Numerical chromosomal instability mediates susceptibility to radiation treatment. Nat. Commun. 6, 5990. 10.1038/ncomms6990 25606712 PMC4516720

[B3] BEIR VII (2006). Health risks from exposure to low levels of ionizing radiation. Washington, DC: National Academy of Sciences.

[B4] Betlazar C. Middleton R. J. Banati R. B. Liu G. (2016). The impact of high and low dose ionising radiation on the central nervous system. Redox Biol. 9, 144–156. 10.1016/j.redox.2016.08.002 27544883 PMC4993858

[B5] Bickmore W. A. van Steensel B. (2013). Genome architecture: domain organization of interphase chromosomes. Cell 152, 1270–1284. 10.1016/j.cell.2013.02.001 23498936

[B6] Bonassi S. Znaor A. Ceppi M. Lando C. Chang W. P. Holland N. (2006). An increased micronucleus frequency in peripheral blood lymphocytes predicts the risk of cancer in humans. Carcinogenesis 28, 625–631. 10.1093/carcin/bgl177 16973674

[B7] Broestl L. Rubin J. B. (2021). Sexual differentiation specifies cellular responses to DNA damage. Endocrinology 162 (11), bqab192. 10.1210/endocr/bqab192 34478502 PMC8462381

[B8] Brown R. E. Freudenreich C. H. (2021). Structure-forming repeats and their impact on genome stability. Curr. Opin. Genet. Dev. 67, 41–51. 10.1016/j.gde.2020.10.006 33279816 PMC8084909

[B9] Bryant P. E. Riches A. C. Terry S. Y. A. (2010). Mechanisms of the formation of radiation-induced chromosomal aberrations. Mutat. Res. 701, 23–26. 10.1016/j.mrgentox.2010.03.016 20348019 PMC6175058

[B10] CCMG-CCGM National Office (2021). CCMG practice guidelines for cytogenetic analysis: recommendations for the indications, analysis and reporting of constitutional specimens (peripheral blood, solid tissues). Available online at: https://www.ccmg-ccgm.org/images/CCMG_practice_guidelines_for_cytogenetic_analysis_B_constitutional_approved_Mar2021.pdf (Accessed January 2, 2025).

[B11] Cherednichenko O. Pilyugina A. Nuraliev S. Azizbekova D. (2024). Persons chronically exposed to low doses of ionizing radiation: a cytogenetic dosimetry study. Mutat. Res. 894, 503728. 10.1016/j.mrgentox.2024.503728 38432778

[B12] Durante M. Formenti S. C. (2018). Radiation-induced chromosomal aberrations and immunotherapy: micronuclei, cytosolic DNA, and interferon-production pathway. Front. Oncol. 8, 192. 10.3389/fonc.2018.00192 29911071 PMC5992419

[B13] Fenech M. (1993). The cytokinesis-block micronucleus technique and its application to genotoxicity studies in human populations. Mutat. Res. 285, 35–44. 10.1016/0027-5107(93)90014-L 7678131

[B14] Fenech M. (2000). The *in vitro* micronucleus technique. Mutat. Res. 455, 81–95. 10.1016/S0027-5107(00)00065-8 11113469

[B15] Fenech M. (2007). Cytokinesis-block micronucleus cytome assay. Nat. Protoc. 2, 1084–1104. 10.1038/nprot.2007.77 17546000

[B16] Feng W. Chakraborty A. (2017). Fragility extraordinaire: unsolved mysteries of chromosome fragile sites. Adv. Exp. Med. Biol. 1042, 489–526. 10.1007/978-981-10-6955-0_21 29357071 PMC6055930

[B17] Gnanasekaran T. S. (2021). Cytogenetic biological dosimetry assays: recent developments and updates. Radiat. Oncol. J. 39, 159–166. 10.3857/roj.2021.00363 34610654 PMC8497872

[B18] Goodarzi A. A. Jeggo P. A. (2013). The repair and signaling responses to DNA double-strand breaks. Adv. Genet. 82, 1–45. 10.1016/B978-0-12-407676-1.00001-9 23721719

[B19] Hastings R. J. Moore S. Chia N. (2024). ISCN 2024: an international system for human cytogenomic nomenclature. Basel: S: Karger.

[B20] Henry E. Arcangeli M.-L. (2021). How hematopoietic stem cells respond to irradiation: similarities and differences between low and high doses of ionizing radiations. Exp. Hematol. 94, 11–19. 10.1016/j.exphem.2020.11.003 33290858

[B21] Holland N. Bolognesi C. Kirsch-Volders M. Bonassi S. Zeiger E. Knasmueller S. (2008). The micronucleus assay in human buccal cells as a tool for biomonitoring DNA damage: the HUMN project perspective. Mutat. Res. Rev. 659, 93–108. 10.1016/j.mrrev.2008.03.002 18514568

[B22] Huang L. Snyder A. R. Morgan W. F. (2003). Radiation-induced genomic instability and its implications for radiation carcinogenesis. Oncogene 22, 5848–5854. 10.1038/sj.onc.1206697 12947391

[B23] IAEA (2014). Protection and safety of radiation sources: international basic safety standards. Vienna: International Atomic Energy Agency.

[B24] ICRP (2007). The 2007 recommendations of the international commission on radiological protection. Ann. ICRP 37, 1–332. 10.1016/j.icrp.2007.10.003 18082557

[B25] ICRP (2017). Annals of the ICRP. London: ICRP Publications.

[B26] INSST (2025). “Ionising radiation,” in Industrial hygiene. Available online at: https://www.insst.es (Accessed May 13, 2025).

[B27] Jackson S. P. Bartek J. (2009). The DNA-damage response in human biology and disease. Nature 461, 1071–1078. 10.1038/nature08467 19847258 PMC2906700

[B28] Jain V. Saini D. Soren D. C. Kumar V. A. Vivek Kumar P. R. Koya P. K. M. (2023). Non-linear dose response of DNA double strand breaks in response to chronic low dose radiation in individuals from high level natural radiation areas of Kerala coast. Genes Environ. 45, 16. 10.1186/s41021-023-00273-6 37127760 PMC10150514

[B29] Ji K. Wang Y. Du L. Xu C. Liu Y. (2019). Research progress on the biological effects of low-dose radiation in China. Dose-Response 17, 155932581983348. 10.1177/1559325819833484 PMC6393828 30833876

[B30] Karmaker N. N. Maraz N. K. M. Islam N. F. (2021). Fundamental characteristics and application of radiation. GSC Adv. Res. Rev. 7, 64–72. 10.30574/gscarr.2021.7.1.0102

[B31] Katsura M. Cyou-Nakamine H. Zen Q. Zen Y. Nansai H. Amagasa S. (2016). Effects of chronic low-dose radiation on human neural progenitor cells. Sci. Rep. 6, 20027. 10.1038/srep20027 26795421 PMC4726121

[B32] Kelsey K. T. Memisoglu A. Frenkel D. Liber H. L. (1991). Human lymphocytes exposed to low doses of X-rays are less susceptible to radiation-induced mutagenesis. Mutat. Res. Lett. 263, 197–201. 10.1016/0165-7992(91)90001-K 1861683

[B33] Kochanova D. Gulati S. Durdik M. Jakl L. Kosik P. Skorvaga M. (2023). Effects of low-dose ionizing radiation on genomic instability in interventional radiology workers. Sci. Rep. 13, 15525. 10.1038/s41598-023-42139-5 37726322 PMC10509213

[B34] Kote-Jarai Z. Salmon A. Mengitsu T. Copeland M. Ardern-Jones A. Locke I. (2006). Increased level of chromosomal damage after irradiation of lymphocytes from BRCA1 mutation carriers. Br. J. Cancer 94, 308–310. 10.1038/sj.bjc.6602912 16404418 PMC2361110

[B35] Lee Y. Jin Y. W. Wilkins R. C. Jang S. (2019). Validation of the dicentric chromosome assay for radiation biological dosimetry in South Korea. J. Radiat. Res. 60 (5), 555–563. 10.1093/jrr/rrz039 31165147 PMC6806015

[B36] Little J. B. (2000). Radiation carcinogenesis. Carcinogenesis 21, 397–404. 10.1093/carcin/21.3.397 10688860

[B37] Mbutu-Austin I. Modarai B. Ainsbury E. Terry S. Y. A. (2025). Potential biomarkers of chronic low-dose radiation exposure for nuclear medicine technologists. Int. J. Radiat. Biol. 101, 453–466. 10.1080/09553002.2025.2470225 40099925 PMC12036530

[B38] Mitchell J. C. Norman A. (1987). The induction of micronuclei in human lymphocytes by low doses of radiation. Int. J. Radiat. Biol. 52, 527–535. 10.1080/09553008714552031 3499407

[B39] Mitelman F. Johansson B. Mertens F. (2007). The impact of translocations and gene fusions on cancer causation. Nat. Rev. Cancer 7, 233–245. 10.1038/nrc2091 17361217

[B40] Monteiro Gil O. Martins J. O. Rosário P. (2020). Use of biological dosimetry to confirm radiation exposure: case study. Radiat. Phys. Chem. 171, 108683. 10.1016/j.radphyschem.2019.108683

[B41] Mothersill C. Seymour C. (2014). Implications for human and environmental health of low doses of ionising radiation. J. Environ. Radioact. 133, 5–9. 10.1016/j.jenvrad.2013.12.018 23664231

[B42] Murmann-Konda T. Soni A. Stuschke M. Iliakis G. (2021). Analysis of chromatid-break-repair detects a homologous recombination to non-homologous end-joining switch with increasing load of DNA double-strand breaks. Mutat. Res. Genet. Toxicol. Environ. Mutagen. 867, 503372. 10.1016/j.mrgentox.2021.503372 34266628

[B43] Narendran N. Luzhna L. Kovalchuk O. (2019). Sex difference of radiation response in occupational and accidental exposure. Front. Genet. 10, 260. 10.3389/fgene.2019.00260 31130979 PMC6509159

[B44] NCRP (2009). Ionizing radiation exposure of the population of the United States. Bethesda, MD: National Council on Radiation Protection and Measurements.

[B45] Norppa H. Falck G. C. (2003). What do human micronuclei contain? Mutagenesis 18 (3), 221–233. 10.1093/mutage/18.3.221 12714687

[B46] Obe G. Pfeiffer P. Savage J. R. Johannes C. Goedecke W. Jeppesen P. (2002). Chromosomal aberrations: formation, identification and distribution. Mutat. Res. 504, 17–36. 10.1016/S0027-5107(02)00076-3 12106643

[B47] Ozkan E. Lacerda M. P. (2023). “Genetics, cytogenetic testing and conventional karyotype,” in StatPearls. Treasure Island, FL: StatPearls Publishing. Available online at: https://www.ncbi.nlm.nih.gov/books/NBK563293/. 33085440

[B48] Piotrowski I. Kulcenty K. Suchorska W. M. Skrobała A. Skórska M. Kruszyna-Mochalska M. (2017). Carcinogenesis induced by low-dose radiation. Radiology Oncol. 51, 369–377. 10.1515/raon-2017-0044 29333114 PMC5765312

[B49] Rall-Scharpf M. Friedl T. W. P. Biechonski S. Denkinger M. Milyavsky M. Wiesmüller L. (2021). Sex-specific differences in DNA double-strand break repair of cycling human lymphocytes during aging. Aging (Albany NY) 13 (17), 21066–21089. 10.18632/aging.203519 34506302 PMC8457596

[B50] Rothkamm K. Löbrich M. (2003). Evidence for a lack of DNA double-strand break repair in human cells exposed to very low X-ray doses. Proc. Natl. Acad. Sci. U. S. A. 100, 5057–5062. 10.1073/pnas.0830918100 12679524 PMC154297

[B51] Schmitz-Feuerhake I. Busby C. Pflugbeil S. (2016). Genetic radiation risks: a neglected topic in the low dose debate. Environ. Health Toxicol. 31, e2016001. 10.5620/eht.e2016001 26791091 PMC4870760

[B52] Shimura N. Kojima S. (2018). The lowest radiation dose having molecular changes in the living body. Dose-Response 16, 1559325818777326. 10.1177/1559325818777326 29977175 PMC6024299

[B53] Smith L. E. Nagar S. Kim G. J. Morgan W. F. (2003). Radiation-induced genomic instability: radiation quality and dose response. Health Phys. 85, 23–29. 10.1097/00004032-200307000-00006 12852467

[B54] Stewart F. A. Akleyev A. V. Hauer-Jensen M. Hendry J. H. Kleiman N. J. MacVittie T. J. (2012). ICRP publication 118: ICRP statement on tissue reactions and early and late effects of radiation in normal tissues and organs—threshold doses for tissue reactions in a radiation protection context. Ann. ICRP 41, 1–322. 10.1016/j.icrp.2012.02.001 22925378

[B55] Topic A. Malic Z. Francuski D. Stankovic M. Markovic B. Soskic B. (2016). Gender-related differences in susceptibility to oxidative stress in healthy middle-aged Serbian adults. Biomarkers 21, 186–193. 10.3109/1354750X.2015.1123287 26754535

[B56] Tubiana M. Aurengo A. Averbeck D. Masse R. (2006). The debate on the use of linear no-threshold for assessing the effects of low doses. J. Radiological Prot. 26, 317–324. 10.1088/0952-4746/26/3/N01 16926474

[B57] UNSCEAR (2020). Sources, effects and risks of ionizing radiation in UNSCEAR 2020 report, vol. I: sources (New York: United Nations).

[B58] Wang W. J. Li L. Y. Cui J. W. (2020). Chromosome structural variation in tumorigenesis: mechanisms of formation and carcinogenesis. Epigenetics Chromatin 13, 49. 10.1186/s13072-020-00371-7 33168103 PMC7654176

[B59] WHO (2023). Health effects of ionizing radiation. Geneva: World Health Organization.

[B60] Wilhelm T. Said M. Naim V. (2020). DNA replication stress and chromosomal instability: dangerous liaisons. Genes 11, 642. 10.3390/genes11060642 32532049 PMC7348713

[B61] Wilkins R. C. Romm H. Kao T. C. Awa A. A. Yoshida M. A. Livingston G. K. (2008). Interlaboratory comparison of the dicentric chromosome assay for radiation biodosimetry in mass casualty events. Radiat. Res. 169 (5), 551–560. 10.1667/RR1272.1 18439045

[B62] Yamashita M. Suda T. (2021). Low-dose ionizing radiations leave scars on human hematopoietic stem and progenitor cells: the role of reactive oxygen species. Haematologica 106, 320–322. 10.3324/haematol.2020.272898 33386716 PMC7776338

